# Effect of Alloying Elements Gradient on Solid-State Diffusion Bonding between Aerospace Aluminum Alloys

**DOI:** 10.3390/ma11081446

**Published:** 2018-08-15

**Authors:** Fan Wu, Wenlong Zhou, Yujie Han, Xuesong Fu, Yanjin Xu, Hongliang Hou

**Affiliations:** 1 School of Material Science and Engineering, Dalian University of Technology, Dalian 116085, China; fusfuxuesong@163.com; 2 AVIC Aeronautical Manufacturing Technology Research Institute, Beijing 100024, China; hanyujiehao@126.com (Y.H.); xuyj_avic@aliyun.com (Y.X.); hou_hl@163.com (H.H.); 3 Key Laboratory of Solidification Control and Digital Preparation Technology (Liaoning Province), School of Materials Science and Engineering, Dalian University of Technology, Dalian 116085, China

**Keywords:** diffusion bonding, interface microstructure, shear strength, alloying element diffusion

## Abstract

Three different bonding couples assembled by two commonly used aerospace aluminum alloys were bonded within the temperature range of 460–520 °C under 6 MPa for 60 min in vacuum atmosphere. The interface microstructure and alloying elements distribution of the bonded joints were determined by scanning electron microscope (SEM) and Energy Dispersive Spectroscope (EDS); the bond strength was evaluated by tensile-shear strength test. The results show the bond quality improved effectively as the bonding temperature increased. Compared with the 1420-1420 and 7B04-7B04 bonding couples, the 1420-7B04 couples obtained better interface integrity and higher bond strength, the highest shear strength for 1420-7B04 couple can be as high as 188 MPa when bonded at 520 °C. Special attention was focused on the 1420-7B04 couple, the diffusion coefficient of Mg at the original interface under different temperatures were investigated, the results show the diffusion coefficient increased obviously as the bonding temperature increased. A diffusion affected zone (DAZ) without continuous intermetallic phases formed due to the diffusion of alloying elements across the bonding interface. The combined action of temperature and alloying elements gradient resulted in the increase of alloying elements diffusion fluxes, which in turn promote the bonding quality through the accelerated shrinkage of interfacial voids.

## 1. Introduction

Diffusion bonding (DB) is a process where two nominally flat surfaces are joined at elevated temperatures under an external pressure for a time ranging from a few minutes to longer [1,2]. Superplastic forming (SPF) produces complex near-net shapes, thus reducing the manufacturing costs and weight. When combined, these two technologies together, give rise to the technology known as DB/SPF to produce components with greater design flexibility as well as considerable weight and cost reduction [3,4,5]. The DB/SPF structure of titanium alloys has been widely used in the aerospace industry [5]. While the aluminum alloys are also commonly used aerospace structural materials, the DB/SPF technology would be attractive for aluminum alloys in the aerospace industry to obtain lightweight structures [6]. However, diffusion bonding of the aluminum alloys is difficult due to the tenacious oxide film which exists on the surface. The oxide film prevents the close contact between base materials and acts as a diffusion barrier of alloying atoms, which results in poor bonding quality [7,8]. Hence, to achieve a sound bond interface for aluminum alloys, it is necessary to remove the surface oxide or at least partially disrupt its continuity.

There is a general agreement that all metals are assumed to bond together if two cleaned surfaces are brought into contact within the range of interatomic force [9]. In order to break the oxide film and get close contact between the aluminum alloy substrates, many attempts have been attempted to produce bonds with significant strength, which including large deformation, high temperature and protective atmosphere. The effect of active alloying elements such as magnesium and lithium during diffusion bonding of aluminum-based alloys has been investigated [9,10,11]. When the pre-bonding surfaces were brought into contact, there were interfacial voids formed due to the surface asperities. During diffusion bonding, the void shrinkage is an important process for achieving high quality joints and is generally ascribed to several physical mechanisms [1,7,12]: (1) plastic flow of materials around voids, including plastic deformation and creep deformation; (2) atomic diffusion, including surface diffusion, interface diffusion and volume diffusion. An increase in temperature will reduce the material yield strength, enhancing the plastic flow of materials, thereby forcing more mass from adjacent regions into voids. Simultaneously, void shrinkage is further accelerated by increased atomic diffusion. The diffusion coefficient *D* is a material property and is the most useful parameter for characterizing the capability of atomic diffusion, which is generally described as follows:

(1) $$D=D_{0}\exp\left(-\frac{Q}{RT}\right)$$

where *D*_0_ (m^2^/s) is the diffusion constant, *Q* (J/mol) is the activation energy for the atomic diffusion, *R* (J/(mol·K)) is the gas constant, and *T* (°C) is the temperature. It is apparent from Equation (1) that the diffusion coefficient is a strong function of temperature, and an increase in temperature enhances the capability of atomic diffusion and will, in turn, accelerate void shrinkage.

In the present study, diffusion bonding with or without alloying elements gradient between aluminum alloys were investigated; three different bonding couples were designed to investigate the influence of the bonding temperature and alloying elements gradient on bonding quality. Two commonly used aerospace aluminum alloys, 1420 alloy and 7B04 alloy were chose to conduct these experiments. The bond quality of joints was assessed through the interface microstructure and bond strength, meanwhile, the alloying elements distribution after bonding was determined to study its diffusion behavior during bonding. The alloying elements gradient was introduced to increase the alloying elements diffusion fluxes across the bonding interface, which in turn improves the bond quality; this is the hypothesis we want to prove in the present study.

## 2. Experimental

**Figure 1:**
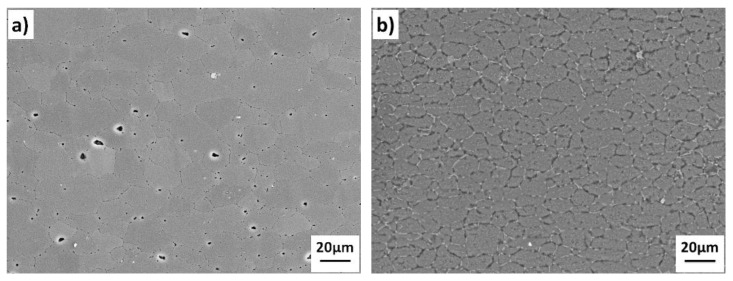
The original microstructure of the base materials: (**a**) 1420; (**b**) 7B04.

**Figure 2:**
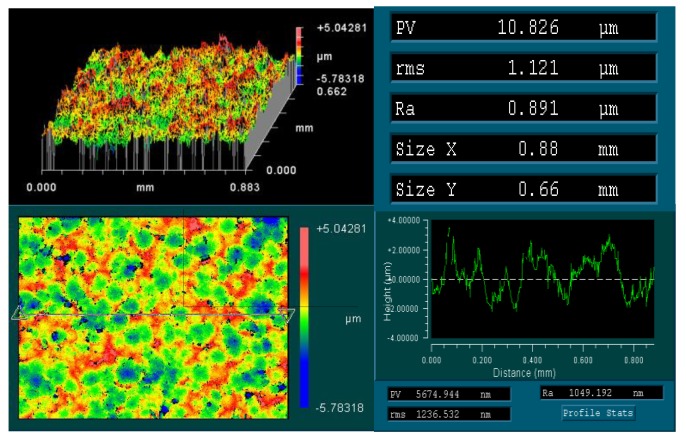
The surface roughness of pre-bonding samples after surface treatment.

The base materials used in the present work were 1420 Al-Li alloy and 7B04 Al alloy, supplied in the form of plates by Central South University (Changsha, China). The chemical composition of base materials used in this study is listed in Table 1. The average grain size of the alloys before bonding were around 10–20 μm for these alloys. Figure 1 shows the as-received base materials demonstrates the original microstructure of base materials. The surface of the sample to be bonded was prepared by mechanical polishing. The surface was polished by 1000 grit SiC paper and degreased with acetone, after that the samples were etched in 10% NaOH for 3 min and then neutralized in 30% HNO_3_ for 5 min at room temperature, then the samples were ultrasonically cleaned in deionized water. The surface roughness of the pre-bonding samples after surface treatment were measured by using a 3D surface profiler (ZYGO-NewView 9000, Zygo Corporation, Berwyn, PA, USA), the results are shown in Figure 2. After the samples cleaned, the specimens were immersed in alcohol immediately to prevent contamination before bonding. Three different diffusion bonding couples (1420-1420 couple, 7B04-7B04 couple and 1420-7B04 couple) were conducted under the same bonding parameters. The bonding process was carried out on Gleeble-3500 thermal simulation testing machine (Dynamic Systems Inc., Poestenkill, NY, USA) at a temperature range of 460–520 °C with 6 MPa for 60 min.

The temperature during the bonding process was measured by a K-thermocouple (OMEGA Engineering Inc., Norwalk, CT, USA) which was welded on the diffusion bonding couple. Before bonding, the diffusion bonding couple was heated to the bonding temperature with a heating rate of 60 °C/min and held at the object temperature for 5 min to eliminate the thermal gradient. After bonding process finished, pressure was released and sample was cooled to room temperature in the work chamber. The diffusion bonding parameters are listed in Table 2. Process parameters such as temperature, pressure and time were automatically controlled during bonding. In order to protect the sample from oxidizing during the bonding process, the work chamber was pumped for vacuum to 1 × 10^−3^ Pa.

**Figure 3:**
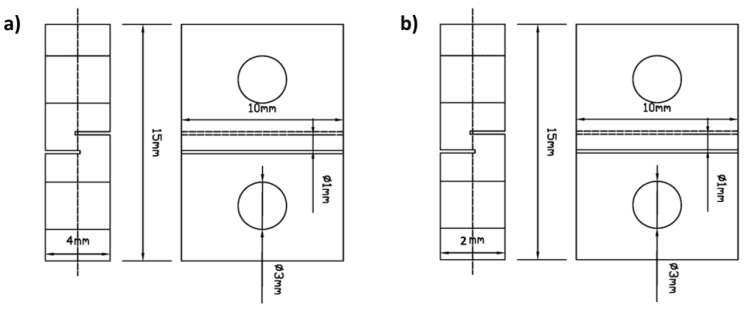
Configuration of shear strength testing pieces (unit: mm): (**a**) For the diffusion bonding joints; (**b**) for the 1420 Al-Li alloy and 7B04 Al alloy base materials.

After the bonding process, the sample was processed to the shear specimens, as the diffusion bonding sample were not large enough for normal shear test, a non-standard shear specimen was devised to measure the shear strength of bond joints according to the results of previous studies [8,13,14]. Shear strength of the bonded joints and base materials were calculated after the shear test, for each bonding condition, seven joints were tested and the average values were taken. The dimension of the shear specimen was given in Figure 3, shear strength test was carried out on microcomputer controlled electronic universal testing machine (WDW-50E, Lubiao Test, Jinan, China) with a crosshead speed of 0.3 mm/min. The interface microstructure and fracture morphology were analyzed by the scanning electron microscope (SEM, ZEISS SUPRA 55, Oberkochen, Germany) equipped with an energy dispersive spectroscope (EDS). The sample was prepared using standard metallographic technique and Keller’s reagent was used for etching.

## 3. Results and Discussion

### 3.1. Shear Strength of the Bond Joints

**Figure 4:**
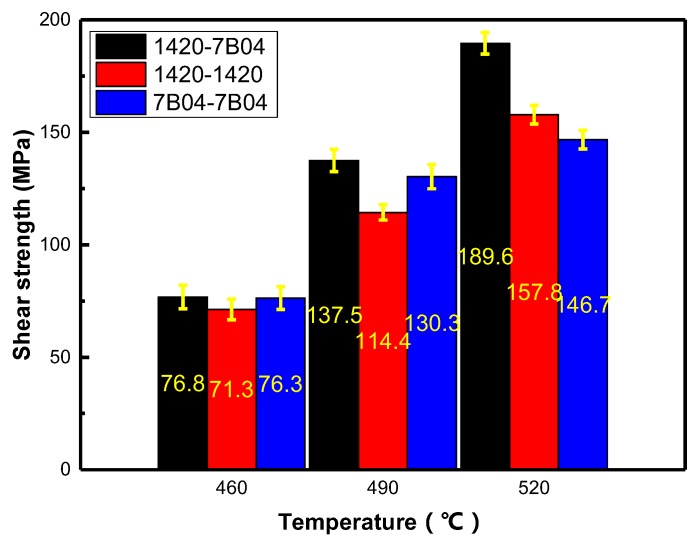
Shear strength of different bonded joints under different bonding parameters.

The shear strength was measured to evaluate the bonding quality, and the average shear strength values of the samples are shown in Figure 4. It can be concluded from the results that the bonding temperature influences the bond strength directly. For the bonded joints of 7B04-7B04 couple, the shear strength values were 76.3, 130.3 and 146.7 MPa corresponding to bond temperature 460, 490 and 520 °C, respectively; and for the bonded joints of 1420-1420 couple, the shear strength values were 71.3, 114.4, 157.8 MPa; and 76.8, 137.5, 189.6 MPa for the 1420-7B04 couple, respectively. The shear strength of the bonded joints and the base materials show that the shear strength values fluctuate to a certain extent, and the deviation of results within the range of ±10 MPa. The results revealed that the increase of shear strength value is proportional to the increase of bonding temperature for all the diffusion bonding couples. This is similar to the investigation made by Liu Liming et al. [15]; in their experimental study, shear strength values of the bonded joint between different metallic materials increased with the bonding temperature.

The minimum shear strength value was observed for samples bonded at 460 °C, the poor shear strength is attributed to the poor contact between the adjacent surfaces and low diffusion coefficients of alloying elements under lower bonding temperature. To further increase the bonding temperature to 490 °C, higher shear strength values were obtained. The maximum shear strength values for all these three different bonding couples was obtained at 520 °C, high bonding temperature leads to high diffusion fluxes of alloying element across the interface and results in good bond quality.

In general, it is difficult to compare the shear strength values with those which have been reported for other Al alloys because of the difference in composition, bond conditions and post-bond heat treatment. The best shear strength measured in the present work was higher than the average value of 150 MPa obtained for comparable Al alloys such as AA7475 [16]. Hence, to exclude these effects, the specific shear strength (*μ*) is used to assess the bond quality [17], which defined as follows:

(2) $$\mu =\frac{\tau_{DB}}{\tau_{B}}$$

Here $\tau_{DB}$ is the shear strength of bonding joint, and $\tau_{B}$ (MPa) is the shear strength of base material, the shear strength of base materials is 166.4 and 206.7 MPa for the 7B04 Al alloy and 1420 Al-Li alloy, respectively. In this study, the shear strength of 1420 Al-Li alloy base material was chose as the $\tau_{B}\;$ to determine the value of, *μ*_1420-1420_ and *μ*_1420-7B04_, and the shear strength of 7B04 Al alloy base material was chose as the τ_B_ to determine the value of *μ*_7B04-7B04_. The corresponding specific shear strength values are shown in Table 3. The results show that the specific shear strength of 1420-7B04 couple can be as high as 0.92 when the bonding under 520 °C.

### 3.2. Interface Characteristics and the Fracture Morphology

**Figure 5:**
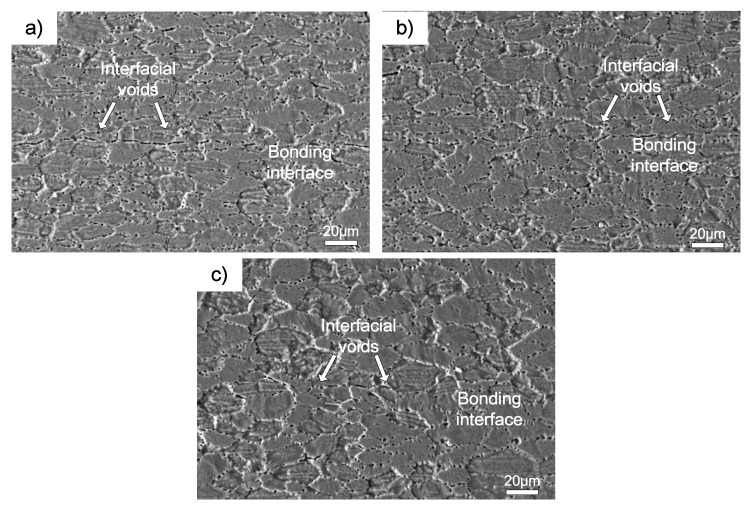
SEM micrographs of the 7B04-7B04 interface microstructure at different temperatures: (**a**) 460 °C; (**b**) 490 °C; (**c**) 520 °C.

It is well known that the bond strength was directly related to the interface integrity and corresponding to elimination of interfacial voids. Microstructural studies were made on samples after shear strength test. Generally, the microstructure evolution is affected by the temperature during bonding. The interface microstructure and the corresponding fracture morphology of the three different bonding couples bonded in the temperature range of 460–520 °C are shown in the following figures. Figure 5 shows the interface microstructure of 7B04-7B04 couple after being bonded at different temperatures. There were clear evidences of continuous interfacial voids existing along the bond interface when bonded at 460 and 490 °C, and the shrinkage of interfacial voids were accelerated as the bond temperature increased. To further increase the bond temperature to 520 °C, the bond interface would have a microstructure indistinguishable from the adjacent parent metal. The results show the metallurgical bond obtained under various temperature, and the metallurgical bond areas increased with the temperature. It can be seen from Figure 5c that a relatively large grain size was obtained when bonded at 520 °C. The grain growth may affect the mechanical properties of joints to some extent according to the previous study [18,19,20]. Meanwhile, there is clear evidence of local migration of grain boundaries leading to formation of triple point junctions across the interface.

**Figure 6:**
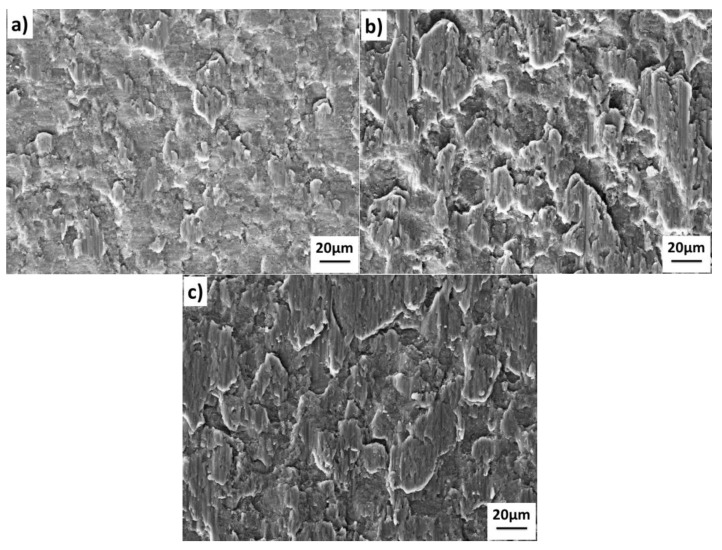
Shear test fractographs of the 7B04-7B04 joints after bonded at different temperatures: (**a**) 460 °C; (**b**) 490 °C; (**c**) 520 °C.

The shear fracture surface of the 7B04-7B04 joints bonded under different temperatures are shown in Figure 6. The fracture morphology shows that the fracture surface of 7B04-7B04 bonded joints contains two kinds of zones, namely, the unbonded zone and the shear band zone. The fracture morphology corresponds to the interface quality in Figure 6. The interfacial voids in Figure 5a corresponds to the unbonded areas in Figure 6a, and the metallic bond areas in Figure 5 correspond to the shear band zone in Figure 6. The results show that the shear band zone increases with the bond temperature and results in higher shear strength.

**Figure 7:**
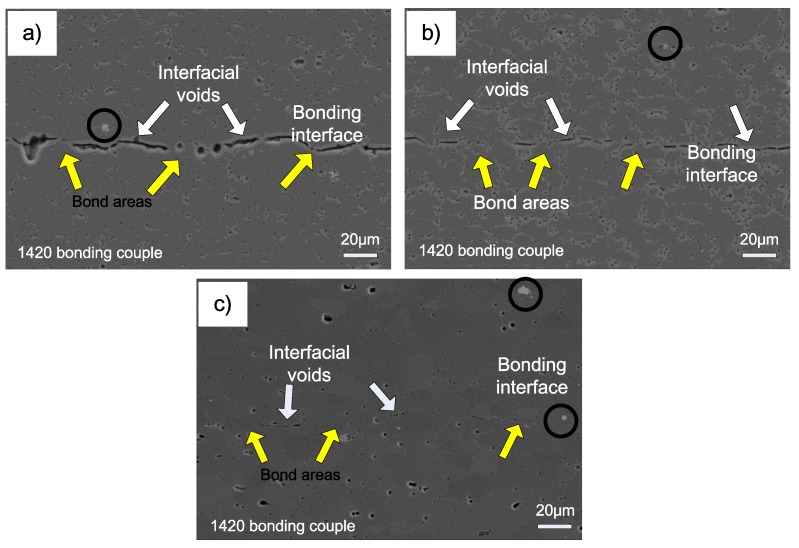
SEM images of the 1420-1420 interface characteristics after bonded at different temperatures: (**a**) 460 °C; (**b**) 490 °C; (c) 520 °C.

Interface microstructure of 1420-1420 couple bonded under various temperatures is shown in Figure 7. The interface microstructure evolution of 1420-1420 couple during bonding is similar to the 7B04-7B04 couple. As seen from Figure 7a, only local areas obtained metallurgical bonding and there are continuous interfacial voids distributed along interface when bonded at 460 °C. When bonded at 490 °C, the dimension of interfacial voids decreased obviously and the metallurgical bonding areas increased evidently. To further increase the temperature to 520 °C, the interfacial voids became round in shape and small in size, and the long interfacial voids disappeared and the bond areas increased significantly. The results show that it is difficult for 1420-1420 couple to obtain sound joints without interfacial voids under the present bonding parameters. This is similar to the results of former study [21], who concluded that it is difficult to produce a joint with 100% metallurgical bond area. Compared with the 7B04-7B04 bond couple, there is no obvious grain growth in the 1420-1420 bond couple. This can be ascribed to the existence of Al_3_Zr phase (as shown in Figure 7c with black circles), which pinned up the grain boundary and inhibited the grain growth [22,23].

**Figure 8:**
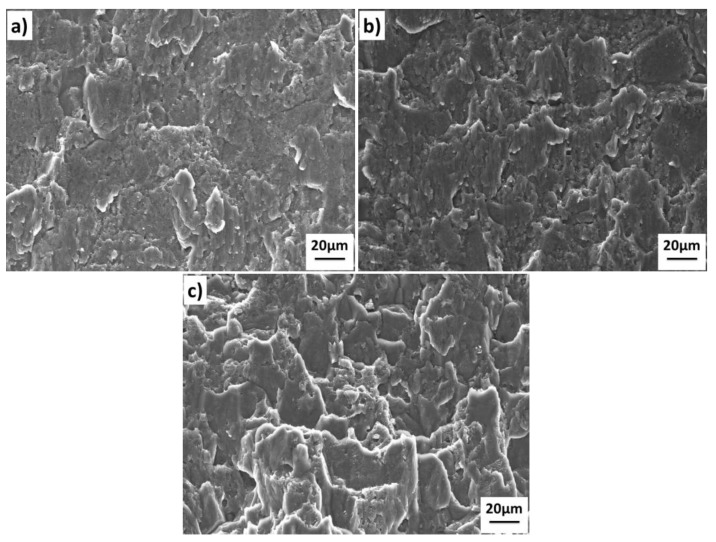
Shear test fractographs of the 1420-1420 joints after bonded at different temperatures: (**a**) 460 °C; (**b**) 490 °C; (**c**) 520 °C.

The shear fracture surface of the 1420-1420 joints bonded under different temperatures are shown in Figure 8. The results show that the fracture morphology corresponds to the interface quality in Figure 7. The interfacial voids in Figure 7 corresponds to the unbonded areas in Figure 8, and the metallic bond areas corresponding to the shear band zone and small dimples at the tip of the shear band. The main difference of the fracture morphology between the 1420-1420 couple and the 7B04-7B04 couple is the existence of the dimples at the tip of the shear band. The results show that the increase of the shear band zone and dimples can improve bond quality with higher shear strength. 

**Figure 9:**
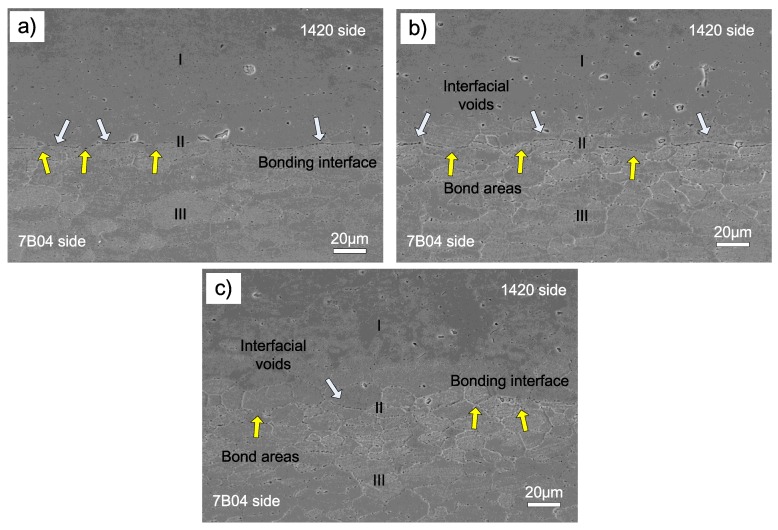
SEM images of the 1420-7B04 interface microstructure after bonded at different temperatures: (**a**) 460°C; (**b**) 490°C; (**c**) 520°C.

The interface microstructure of 1420-7B04 couple after bonded at different temperature was shown in Figure 9. When bonded at 460 °C, the long and continuous voids exist along the interface, only a limited areas obtain metallic bond along the bonding interface, as shown in Figure 9a. As the bonding temperature increased to 490 °C, it is obviously, by comparing Figure 9a,b, the voids size decreased and the metallic bond areas increased. To further increase the bond temperature to 520 °C, the interfacial voids can be seldom found and the original bond interface is similar to the adjacent grain boundaries. The results show that the metallic bond areas increased with the bond temperature rose, which is similar to the 1420-1420 couple and 7B04-7B04 couple. Nevertheless, the interface microstructure of the 1420-7B04 couple is quite different from the 1420-1420 couple and 7B04-7B04 couple, that’s because the alloying element gradients actuated the mutual diffusion across the bonding interface and resulted in the formation of diffusion affected zone (DAZ) formed. The DAZ divided the bond interface into three zones which are denoted as I, II and III and represent the regions 1420 base metal, DAZ and 7B04 base metal, respectively. For the 7B04 side, the grain in the DAZ region is smaller than the grains of 7B04 substrate, while on the 1420 side, there is no obvious change of grain size under the present bond parameters. When the bond temperature increased from 460 to 520 °C, the bond areas and the diffusion flux of alloying elements across the interface increased, meanwhile the thickness of DAZ increased obviously (Figure 9), the diffusion behavior of alloying elements and the thickness of DAZ will be discussed in the following part.

**Figure 10:**
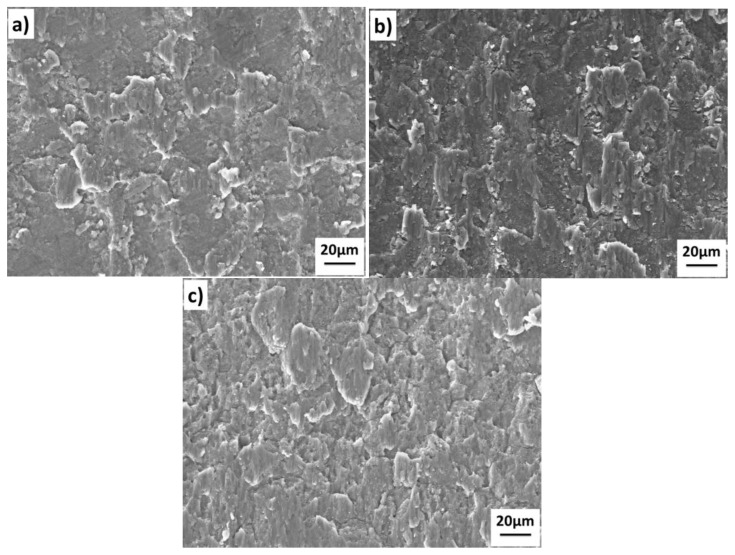
Shear test fractographs of the 1420-7B04 joints after bonded at different temperatures: (**a**) 460 °C; (**b**) 490 °C; (**c**) 520 °C.

The shear fracture surface of the 1420-7B04 joints bonded under different temperatures is shown in Figure 10. The interfacial voids in the Figure 9 corresponding to the unbonded areas in Figure 10, and the metallic bond areas corresponds to the shear band zone and dimples across the fracture surface. The fracture morphology of the 1420-7B04 bonded joints show that the fracture surface contains the unbonded zone, the shear band zone and large dimples. The main difference of the fracture morphology between the 1420-7B04 couple and the 1420-1420 couple are that the number and size of the dimple increased obviously. The fracture surfaces of the 1420-7B04 bonded joints show different morphology compared with the 1420-1420 bonded joints and 7B04-7B04 bonded joints; this can prove that the fracture location is in the DAZ. The different fracture morphology of the 1420-7B04 bonded can be ascribed to the evolution of alloying elements distribution and microstructure in the DAZ. The alloying elements diffusion leads to the microstructure evolution around the bonding interface and the formation of large dimples in the fracture surface, which results in high bond quality for the 1420-7B04 couple.

### 3.3. Diffusion Behavior of the Alloying Elements

In order to analyze the diffusion of alloying elements, the mass concentration distribution after diffusion bonding was investigated. For bond joints of 1420-1420 couple and 7B04-7B04 couple, as the alloying element concentration across the interface is similar, the alloying atoms diffuse in short distance by means of “random walk” around their initial position. Once the surface atoms move close enough to form metallic bond, the bond areas increase with the local plastic deformation and diffusion of alloying elements during the bond process. For the bond couple of 1420-7B04, the diffusion of alloying atoms is somewhat different from that in the homogeneous region because the concentration gradient of alloying elements exists in macroscopic scale, such as Zn and Mg, thus the “downhill atomic diffusion” occurs. The diffusion of alloying elements induces a decrease in alloying elements concentration gradient between base materials, which resulted in the formation of DAZ with a certain width and determined the bond quality to some extent. In the present study, the 1420 Al-Li alloy enriched with Li and Mg solutes, while the 7B04 Al alloy primarily contained Zn and Cu solute atoms. According to the Fick’s law and the alloying elements composition across the bond interface, the Mg and Li atoms diffuse from the 1420 side to 7B04 side, while the Zn and Cu atoms move in the opposite direction. To illuminate the formation of DAZ and diffusion behavior of alloying elements, the distribution of alloying elements across the bond interface must be confirmed. However, the EDS could not readily detect Li atom; meanwhile, the mass concentration of Cu atoms is low. The alloying elements Mg were selected as the primary alloying elements to investigate in this study for clarity. Figure 7 shows the concentration profiles of Mg and Zn under different bonding temperature. The mass concentration of Mg decreased gradually across the interface from the 1420 to 7B04. The Mg content was approximately 5.0 wt.% on the 1420 base material, and this value decreased to approximately 2.0 wt.% of the 7B04 substrate. Hence, the thickness of DAZ was defined as the distance across the interface over which the Mg decreased from the stable value approximately 5.0 wt.% on 1420 side to the stable value 2.0 wt.% on 7B04 side.

The diffusion behavior of alloying elements can be analyzed by the following equations [24,25,26], the diffusion flux of alloying elements was determined by the alloying element concentration gradient across the bonding interface and temperature according to the Equation (3):

(3) $$J=-D\frac{dC}{dx}$$

Here *J* is the diffusion flux; *D* is the diffusion coefficient; d*C*/d*x* is the concentration gradient. To elevate the diffusion fluxes of alloying elements across the bond interface, the diffusion coefficient should be examined firstly. The diffusion of alloying elements in the DAZ is non-steady, meanwhile the mass concentration at a certain point varies with the time. Before bonding, the elements concentration in the both sides of substrate are constant, and the concentration near the interface are abruptly changed. After the diffusion bonding was conducted, the alloying elements concentration in the DAZ is gradually distributed. According to the previous study [27], this process is belonged to non-steady diffusion and the Fick’s second law was followed. The equation for a one dimensional diffusion process is designated and can be expressed as the following:

(4) $$\frac{\partial C}{\partial t}=D\frac{\partial^{2}C}{\partial x^{2}}$$

where *C* is the alloying elements concentration (wt.%), *D* is the diffusion coefficient (μm^2^/s), *t* is holding time (s), *x* is the diffusion distance (μm). As the width of the DAZ is much smaller than the entire specimen, the specimens can be seen as infinite diffusion couples and the initial conditions are:

(5) $$\{\begin{array}{c} C=C_{1},\;x<0\\ C=C_{2},x>0 \end{array},\;t=0$$

where *C*_1_ and *C*_2_ are the alloying elements concentrations in the dissimilar base materials. The Boltzmann-Matano method is used to solve this diffusion problem [28]. The *η* is introduced in the following:

(6) $$\eta =\frac{x}{\sqrt{t}}$$

the equations can be rewritten as:

(7) $$-\frac{\eta }{2}dC=d\left(D\frac{dC}{d\eta }\right)$$

The initial condition can be correspondingly changed to:

(8) $$\{\begin{array}{c} C=C_{1},\;\eta =-\infty \\ C=C_{2},\eta =+\infty \end{array}$$

The elements concentration curve of Mg after diffusion bonding is shown in Figure 7. To determine the diffusion coefficient *D* for any value of concentration *C*_0_ (*C*_1_ < *C*_0_ < *C*_2_), Equation (4) is integrated on both sides:

(9) $$-\int_{C_{1}}^{C_{0}}\frac{\eta }{2}dC=\int_{C_{1}}^{C_{0}}d\left(\frac{dC}{d\eta }\right)={\left(D\frac{dC}{d\eta }\right)}_{C=C_{1}}^{C=C_{0}}={\left(D\frac{dC}{d\eta }\right)}_{C=C_{0}}-{\left(D\frac{dC}{d\eta }\right)}_{C=C1}$$

In the above equation, there is the following relationship:

(10) $$D\frac{dC}{d\eta }=D\frac{dC}{dx}\frac{dx}{d\eta }=D\sqrt{t}\frac{dC}{dx}$$

Combining the equations above, the following equation is obtained:

(11) $$D\left(C_{0}\right)=-\frac{1}{2t}{\left(\frac{dx}{dC}\right)}_{C=C_{0}}\int_{C_{1}}^{C_{0}}xdC$$

**Figure 11:**
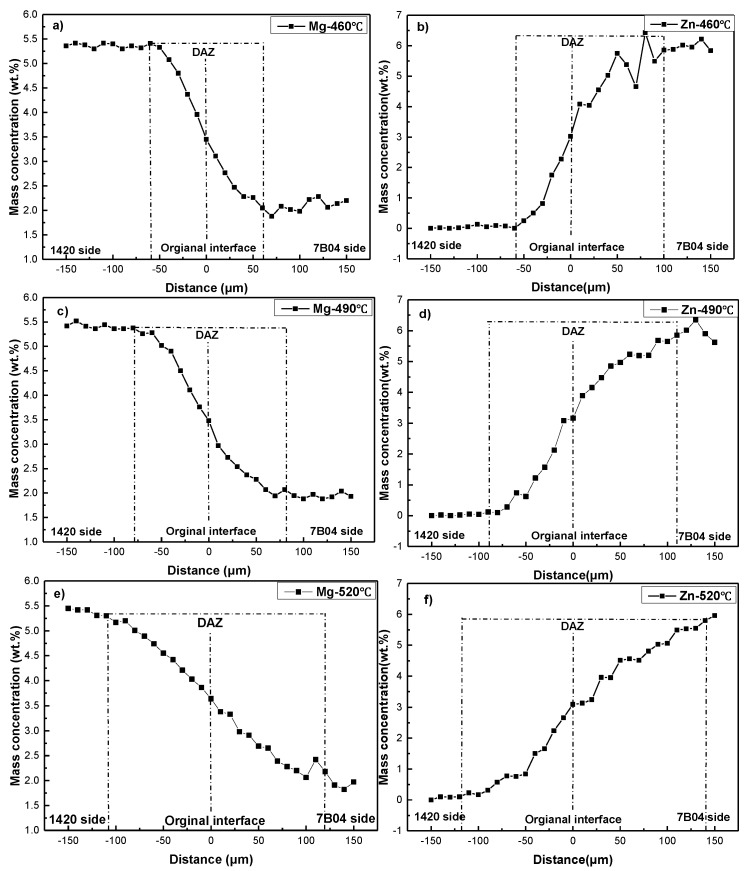
The distribution of main alloying elements (Mg and Zn) across the interface after bonded under different temperatures: (**a**,**b**), 460 °C; (**c**,**d**), 490 °C; (**e**,**f**), 520 °C.

Equation (11) reveals a quantitative relationship between diffusion coefficient *D* and concentration *C* at different positions in the DAZ, in the present study, the *C*_1_ is 2.0 wt.%, while the *C*_2_ is 5.0 wt.%. The diffusion coefficient of Mg from the 1420 side to 7B04 side can be calculated according to the elements concentration distribution in the Figure 11 and the Equation (11), and the diffusion coefficient of Mg at the original interface have been calculated and listed in Table 4. The results show the diffusion coefficient of Mg increased obviously as the bonding temperature increased from 460 to 520 °C. According to the Equation (2), it can be concluded that the increase of *D* and the alloying elements concentration gradients can elevate the diffusion fluxes of alloying elements across the bonding interface, which in turn, accelerated the shrinkage of the interfacial voids and improved the bond interface integrity.

Figure 11 shows that the thickness of DAZ was approximately 120, 160 and 230 μm corresponding to the bonding temperature of 460, 490 and 520 °C, respectively. The formation of DAZ can be ascribed to the different diffusion distance of alloying elements under different bonding temperature, the results show the thickness of DAZ increased as the bonding temperature rose. Meanwhile, according to the alloying elements distribution across the bond interface (as shown in Figure 11) and interface microstructure (as shown in Figure 9), no evidence of continuous intermetallic compound was found throughout the interface, which indicated excellent metallurgical bonding between the 1420 and 7B04 alloy. In a similar study, when diffusion bonding was conducted between two Al and Mg, there were intermetallic layers obtained along the bond interface which was detrimental to the bond strength and caused brittle fracture during the shear strength test.

### 3.4. Discussion

In the present study, diffusion bonding between aerospace aluminum alloys with an alloying elements concentration gradient were conducted and investigated in detail. The effect of alloying elements gradient on the bond quality was analyzed in detail through the comparison of interface microstructure and bond strength between the diffusion bonding couples with or without alloying elements gradients. The alloying elements diffusion was investigated through the detection of alloying element distribution after bonding. The results suggest that the bond strength is strongly influenced by the bonding temperature and the alloying elements diffusion. The role of alloying elements diffusion in producing the bond joints was assessed in the present study. To create a metallic bond, the bond surfaces must be sufficiently close to each other in order to activate inter-atomic short range forces and generate metallic bond joints through diffusion. During the diffusion bonding process, a sound interface without defect obtained depends on the following mechanisms: plastic deformation due to the surface asperities in the initial stage to obtain intimate contact between the pre-bonding surface; the surface-source diffusion mechanism is dominant in the following stage to change the shape of interfacial voids; the interface source mechanism and creep deformation mechanism manners to the interfacial voids elimination. Meanwhile, the bonding surfaces must be free of any contamination, while the oxides on aluminum alloys are physically very sticky and chemically very stable and insoluble in aluminum even at high temperatures [29,30]. Therefore, it makes it difficult to fulfill the full metal-to-metal joints when diffusion bonding is conducted between aluminum alloys. The main way to overcome the problem of oxide films in diffusion bonding is to use relatively rougher surfaces, which causes local plastic deformation in the early stages of bonding and leads to the rupture of oxide film. When the rupture occurs in the oxide film, the physical contact between the pre-bonding surfaces obtained, meanwhile the diffusion paths of alloying elements were established.

In the present study, the formation of bond joints between 1420 Al-Li alloy and 7B04 Al alloy could be described as follows: (1) due to the different plasticity between aluminum substrates and surface oxide films, oxide film breaks resulted firstly due to local plastic deformation and power-law creep deformation caused by the surface asperities; (2) then the substrate comes into contact, the metal-to-metal bonds formed when two physically clean surfaces are brought into contact and the diffusion paths were established; (3) because the alloying elements concentration gradient exists across the bonding interface, the alloying elements diffusion was actuated and accelerated the shrinkage of the interfacial voids, and the metallic bond areas increased; (4) finally, the joint interface integrity increases when the diffusion fluxes of alloying elements increased and the bonding quality was improved.

## 4. Conclusions

In this study, the influence of alloying elements concentration gradient on diffusion bonding between aluminum alloys was investigated. Some main conclusions can be drawn as follows:

- The results show that the shear strength of bond joints increased as the bonding temperature increased. The maximum shear strengths were 157.8, 146.7 and 189.6 MPa when bonded under 520 °C for the three diffusion bonding couples 1420-1420 couple, 7B04-7B04 couple and 1420-7B04 couple, respectively; the specific shear strength results show that the optimal bond parameter for diffusion bonding of the 1420-7B04 couple was 520 °C under 6 MPa for 60 min.
- The diffusion coefficient of Mg increased with the increase of bond temperature, combined the increased diffusion coefficient with the alloying elements gradient, the diffusion fluxes of alloying elements increased obviously, which in turn, improved the interface integrity and bond quality obviously when compared with the 1420-1420 couple and 7B04-7B04 couple.
- Comparing the 1420-7B04 bond couples with the 1420-1420 couple and 7B04-&B04 couple, the former obtained higher interface quality and bond strength under the same bond parameters, which is achieved by increasing the alloying element diffusion flux in the interface region. Meanwhile, the thickness of DAZ increases with the temperature increases, the thickness of DAZ can be as high as 220 μm when bonded at 520 °C.

## Figures and Tables

**Table 1:** Chemical composition of parent metals (wt.%).

Alloys	Zn	Mg	Li	Zr	Cu	Fe	Si	Al
1420	–	5.2	2.0	0.12	0.03	0.07	0.03	Bal.
7B04	5.6	1.9	–	–	1.3	0.05	<0.10	Bal.

**Table 2:** Diffusion bonding parameters and bonding couples conducted in the present study.

Samples	Diffusion Bonding Couple	Temperature (°C)	Pressure (MPa)	Holding Time (min)
1	1420-1420	460	6	60
2	7B04-7B04	460	6	60
3	1420-7B04	460	6	60
4	1420-1420	490	6	60
5	7B04-7B04	490	6	60
6	1420-7B04	490	6	60
7	1420-1420	520	6	60
8	7B04-7B04	520	6	60
9	1420-7B04	520	6	60

**Table 3:** Specific shear strength of the three diffusion couples bonded under different temperatures.

Specific Shear Strength	460 °C	490 °C	520 °C
*μ*_1420-1420_	0.34	0.55	0.76
*μ*_7B04-7B04_	0.46	0.78	0.88
*μ*_1420-7B04_	0.37	0.67	0.92

**Table 4:** Diffusion coefficient of Mg at the original interface under different bonding temperatures.

Temperature (°C)	*D*_Mg_ (m^2^/s)
460	1.015 × 10^−12^
490	1.346 × 10^−12^
520	2.738 × 10^−12^
